# Review of "Introduction to instrumentation and measurements"

**DOI:** 10.1186/1475-925X-5-9

**Published:** 2006-02-08

**Authors:** Max E Valentinuzzi

**Affiliations:** 1 Fellow Investigator, Department of Bioengineering, Instituto Superior de Investigaciones Biológicas (INSIBIO), Universidad de Tucumán/Consejo Nacional Investigaciones Científicas, 4000 Tucumán, Argentina

When Francisco Azuaje, in charge of the Reviews Section of *Biomedical Engineering On-Line*, proposed this book to the Editorial Board, I had immediately the inner feeling that the job was worthwhile. When the copy reached me and I browsed it, I said to myself, "boy, this is good!", and I took it with me to Havanna, Cuba, and thereafter, to Natal, Brazil, where I had several previously scheduled activities. Thus, tropical heat and refreshing sunset sea breezes surrounded its reading so offering a nice environment for quietly thinking and rethinking the statements made herein.

It is mandatory to start this review with a hearty and sincere congratulation to the author, Dr. Robert B. Northrop, for a thorough, solid and impressive piece that actually deserves to be called an *opera*, in the best sense of the old Latin meaning of the word, so much, that the word "introduction" in its title appears as a too modest understatement and, perhaps, it ought to be deleted in future editions, to place the whole work in a proper and better perspective. The book contains enough material (supported by excellent mathematics) to sustain more than a single course in instrumentation and measurements, including *Biomedical Engineering*, at medium to advanced levels, better if led by well-versed and experienced teachers, having at hand good laboratory facilities to actually put into practice at least some of its abundant proposals. A life devoted to creative, careful and many times ingenuous and difficult achievements (as, for example, when estimating blood glucose by reflecting a beam of polarized light off the front surface of the ocular lens and measuring the very small optical rotation resulting from the presence of such substance in the aqueous humor) has been obviously condensed in these pages by Dr. Northrop. No doubt, it is a must in any personal and/or engineering laboratory library, be it for advanced undergraduates, graduate students as also for the practicing professional and researcher. Besides, the book has a superb list of references for further study and is clearly written along with readable equations and figures.

The book is composed of 11 high density chapters, each with a summary and a good collection of suggested problems at the end, but without giving the answers nor offering hints for their solutions although, in the Preface, the author asserts that they are solvable and student-tested. Let us briefly go over them pointing their salient features.

Chapter 1, **Measurements systems **(34 pp, 9 problems), calls certainly the attention because, in an area that many people still think as immutably frozen in the Old Days, it deals with the most recent concepts and definitions, as for example the present international standard for the volt, adopted in 1990 and based on a quantum phenomenon that gives rise to the *Josephson Junction *(JJ), which, when irradiated with microwave energy, produces a precisely known dc voltage across it. Another well described and backed newly adopted standard is for the ohm, based on the *Quantum Hall Effect *(QHE), from which the *Quantum Hall Resistors *(QHR) arise, as for example R_H_(1) = 25,812.81 ohms = 1 von Klitzing (new unit named after the physicist to first describe the effect in 1980). A third electrical parameter with new insights is capacitance: Probably the most accurate means of measuring it is by comparison with a *calculable capacitor*, as predicted by the electrostatic *Thompson and Lampard theorem*, developed in 1956, and technologically improved later on by Delahaye, Fau, Domínguez and Bellon, in 1987, to establish a standard for the Farad (and also for the QHR). The chapter goes finally into inductance (the least pure of all electrical quantities), time and frequency, mass, length and temperature. Quite sensibly, the author underlines that electrical and physical standards, far from being a close subject, are constantly under development and are changing with advances in quantum physics, laser technology and other pieces of scientific knowledge. Further, he poses questions that may mean revision of the current Standard International (or SI) unit system.

Chapter 2, **Analog signal conditioning **(73 pp, 18 problems), probes into the manipulations performed on the signal between the input transducer and the display, processing and storage systems. All of the latter, as a whole, can be called a recording channel (which is a term common in *Biomedical Engineering *and *Physiology*). Its content is more conventional, but still deep and fresh in many respects covering differential amplifiers, operational amps, analog active filters with op-amps, instrumentation amplifiers, nonlinear analog signal processing by op-amps and special function modules, charge amplifiers (rarely, if ever mentioned in the literature) and phase sensitive rectifiers. The last sentence found in page 105, "use should be made of computer aided circuit analysis in reaching the solutions to the suggested 18 problems", clearly indicates the background required and the level of the book.

Chapter 3, **Noise and coherent interference in measurements (**51 pp, 13 problems), definitely can be used for a one-month special course of 6 to 8 hours per week. As the author says in the chapter summary, it is a heuristic (i.e., it encourages the reader to proceed on his/her own, discovering and rediscovering) yet mathematically sound treatment of this difficult and elusive subject. It starts with random noise in circuits, propagation of gaussian noise through linear filters, broadband noise factor and noise figure of amplifiers, spot noise factor (F_spot_) and figure (which are less commonly found in the literature), transformer optimization of amplifier F_spot _and output SNR (signal to noise ratio), cascaded noisy amps, calculation of the noise limited resolution of certain conditioning systems, modern low noise amplifiers for use in instrumentation signal conditioning systems and coherent interference and its minimization (quite a section the latter, indeed, considering itchy subjects such as transient voltage suppressors or grounding arrangements).

Chapters 4 and 5 deal, respectively, with **DC and AC null measurements **(14 pp, 8 problems; 20 pp, 13 problems). Bridge circuits are essential tools in this highly precise type of measurements and the author does not conceal information nor save any effort in putting over the table all that is needed: For dc, the traditional Wheatstone, the Kelvin specialized arrangement to measure very low resistances, the Anderson constant current loop developed in 1992 for measuring resistance changes in remote resistive sensors and the good old potentiometer. For ac, to measure capacitance, capacitor dissipation factor (D), inductance, inductor quality factor (Q), mutual inductance and the small signal transconductance (g_m_) of bipolar junction transistors (BJT) and field effect transistors (FET), the chapter introduces a nice collection of not frequently found bridges, as the resistance ratio bridge, the Schering bridge, the parallel C bridge, the De Sauty bridge, the Wien bridge, the commutated capacitor bridge, the Maxwell bridge, the parallel inductance bridge, the Hay bridge, the Owen bridge, the Anderson bridge, the Heaviside mutual inductance bridge and the Heydweiller mutual inductance bridge, each with well discussed advantages and disadvantages for their respective specific uses.

Chapter 6, **Survey of sensors input mechanisms **(114 pp, 22 problems), as the title anticipates (a survey), is a long, heavily loaded piece that in itself, as with a previous chapter, covers enough material to implement a 6 to 8 weeks (at 6 to 8 hours a week) course, also well suited for Biomedical Engineering students. The sensor senses the signal and the transducer translates it into another type of energy. By and large, the sensor is also part of the transducer and mostly so much embedded in it that physical separation becomes extremely difficult if not impossible. Several resistive sensing principles are described, among which the *giant magnetoresistive *(GMR) one calls the attention, as a new effect discovered in the late 1980's independently by Peter Gruenberg, in Germany, and Albert Pert, in France. The phenomenon of anisotropic magnetoresistance (AMR), originated by Lord Kelvin in 1856 and revived due to new technologies in 1999, is attractively introduced in good detail. Thereafter, a 20 pages section is devoted to voltage generating sensors, such as the traditional thermocouples, photovoltaic cells and piezoelectric transducers followed by less known types, as pyroelectric ones (substances that produce electrical charges in response to internal heating) and those which depend on magnetic flux time changes (variable reluctance pieces, electrodynamic accelerometers, Faraday effect flowmeters and Hall effect sensors).

The well-known linear variable differential transformer (LVDT), synchros and resolvers belong to the class of sensors based on variable magnetic coupling, the two latter used to detect angular position, which are important in robotics and in myoelectric and neuroelectric prostheses applications. They are treated in Section 6.5, followed in Section 6.6 by variable capacitance sensors and, immediately thereafter, by the attractive and well-updated fiber optic types, which were developed primarily for broadband, long distance communications links. There are three sections dealing, respectively, with the always used photomultipliers, ionizing radiation sensors and electrochemical ones. A novelty in texts of this kind are the electronic "noses", which include some background olfactory physiology, and mechano-optical sensors to close the chapter. In summary, the survey focuses on the more important principles whereby physical quantities (including physiological variables) are converted to electrical signals. The listing is by no means exhaustive, as recognized by the author.

At this point, the beginning of Chapter 7, **Applications of sensors to physical measurements **(150 pp, 10 problems), the book is roughly halfway through. The purpose here (the longest chapter) is to examine the most commonly employed sensor types used to measure a given quantity. The variables considered are acceleration, velocity and position (angular and rectilinear), temperature, pressure, force, torque, gyroscopic measurement of angular velocity and position, the Global Positioning System (GPS) and an introduction to spectrophotometry. The previous chapter, instead, concentrates just on transducer mechanisms. The accurate measurement of very small displacements, as the case is with the eardrum, by means of Fiber Optic Fizeau Interferometry, appears as particularly interesting for it was applied to a common cricket subject to external audible sound. The spectrum covered is very ample and the reader almost certainly will select those specific subjects that better suit his/her interests.

Already within the last third of the book, **Basic electric measurements **are defined in Chapter 8 (88 pp, 12 problems) of the traditional electrical parameters of voltage, current, electric field strength, magnetic fields, resistance, capacitance, inductance, the ac steady state parameters of impedance and admittance, power, frequency and phase. This material is essential for those engineers or scientists involved in metrology and obviously complements the information offered in the previous chapters. Not much further comment can be added here except that the author keeps faithful to his already proved thoroughness and good quality.

**Digital Interfaces and Signal Conditioning **(Chapters 9 and 10, respectively, with 63 pp, 10 problems, and 36 pp, 11 problems) could not lack considering the current and growing technological trends. It begins with the *Sampling Theorem*, Nyquist criterion and the concept of *aliasing *followed by quantization noise and *dithering*, the latter being a method for statistically reducing the quantization errors and harmonic distortion inherent in the ADC (analog to digital conversion) process. It proceeds, thereafter, with digital-to-analog converters (DAC), the hold operation and ADC different types (such as floating point, delta-sigma and data acquisition cards, the latter implemented by many manufacturers as plug-in interface boards for PC's and laptops that allow one or more channels of external analog data to be sampled and digitized). The final 5 sections of the chapter (34 pp) are devoted to very specific and strictly technological subjects: The IEEE-488 Instrumentation Bus or GPIB (no definition is given for the acronym), serial data communications links, the CAMAC (IEEE-583) Modular Instrumentation Standard (again the acronym is not spelled out) and the VXI Modular Instrumentation Architecture, effect of transmission lines on the transfer of digital data, and closing with data transmission on fiber optic cables (FOC). Personally, and with due respect, I have my doubts about the real need of including such material, full of jargon and technologies that may become obsolete at any moment superseded by others. The practitioner involved in the daily know-how usually looks for this kind of information directly from the factory, catalogs, INTERNET or a more experienced colleague. Chapter 10 starts with a review of the z-transform, it continues with a few digital signal processing algorithms, the fast Fourier transform and digital routines for interpolating discrete data. I view it as a handy complement because the reader, in all likelihood, is already familiar with such subjects.

And we reach the final Chapter 11 (some kind of strawberry on top of the cream) entitled **Examples of the Design of Measurement Systems **(29 pp), where four novel systems are described: a microdegree polarimeter for D-glucose in bioreactors, a locator of discharges caused by insulation faults on coaxial power cables, a pulsed laser velocity and range measuring system, and a fourth that illustrates the use of feedback to automatically null systems applied to detect very small changes in capacitance.

To close the review, as a well self-respected grouchy old guy, let me secrete a few **minor complains **based on the premise that perfection, at best, and similar to its mathematical cousin, is an unreachable limit. Perhaps, the book overdoes its purpose a little by dealing with too many subjects. Acronyms have established themselves as integral and bothersome part of our scientific and technical literature, for writing the whole name every time it is needed becomes inconvenient; in opposition, a sentence or a paragraph may turned out to almost incomprehensible if too many acronyms appear in it. My suggestion is to repeat the definition interspersed in the text in order to help its reading. Moreover, I have the impression that a few acronyms skipped their introduction (as NIST, in page 27, and JFET, in page 120, or maybe I did not find them). Returning to the problems, it would have helped the reader if at least every other one of them had at least some lead for its solution, or may be the straight solution. To revise in future editions, I think there are two syntactic errors, one in page 115, first line of paragraph 3.2.2, and another in page 139, second line of paragraph 3.10. Besides, in page 164, second line of Problem 3.1, there is a missing period between "300 K" and "Use 4 kT".

However, do not pay too much attention to the previous paragraph because all that is listed there is really minor and I have already strongly recommended the book in my department as I do it above at the very beginning of the review. Congratulations, once more, Dr. Northrop!

